# Differential expression and correlation of immunoregulation related piRNA in rheumatoid arthritis

**DOI:** 10.3389/fimmu.2023.1175924

**Published:** 2023-05-30

**Authors:** Ruyu Ren, Huiling Tan, Zhuochun Huang, Yuanyi Wang, Bin Yang

**Affiliations:** ^1^ Department of Laboratory Medicine, West China Hospital, Sichuan University, Chengdu, China; ^2^ Department of Spine Surgery, The First Hospital of Jilin University, Changchun, China

**Keywords:** piRNA, PIWI, rheumatoid arthritis, peripheral blood, biomarker

## Abstract

**Background:**

Although PIWI-interacting RNAs (piRNAs) have recently been associated with germline development and many human diseases, their expression pattern and relationship in autoimmune diseases remain indistinct. This study aimed to investigate the presence and correlation of piRNAs in rheumatoid arthritis (RA).

**Methods:**

We first analyzed the expression profile of piRNAs using small RNA sequencing in peripheral leukocytes of three new-onset untreated RA patients and three healthy controls (HCs). We then selected piRNAs related to immunoregulation by bioinformatics analysis and verified them in 42 new-onset RA patients and 81 HCs by RT-qPCR. Furthermore, a receiver operating characteristic curve was generated to quantify the diagnostic performance of these piRNAs. A correlation analysis was conducted to observe the link between piRNA expression and RA clinical characteristics.

**Results:**

A total of 15 upregulated and 9 downregulated piRNAs among 1,565 known piRNAs were identified in peripheral leukocytes of RA patients. Dysregulated piRNAs were enriched in numerous pathways related to immunity. After selection and validation, two immunoregulation piRNAs (piR-hsa-27620 and piR-hsa-27124) were significantly elevated in RA patients and have good abilities to distinguish patients from controls, which have the potential to serve as biomarkers. PIWI and other proteins implicated in the piRNA pathway were also associated with RA.

## 1 Introduction

Rheumatoid arthritis (RA) is a chronic inflammatory autoimmune disease that will bring about accumulating joint damage and irreversible disability (1); therefore, timely and precise diagnosis is of high importance in RA treatment to prevent adverse clinical endpoints. Typically, RA is often diagnosed by elevated anti-cyclic citrullinated peptide antibodies (ACPAs), rheumatoid factor (RF), and other specific autoantibody markers (2). Although the specific pathogenesis remains unclear, it is believed that RA has a strong genetic component (3). For this reason, the identification of novel genetic biomarkers and the integration of multiple indicators may provide better diagnostic insights for clinicians.

Recently, a great number of studies have shown that non-coding RNA (ncRNA), as an important gene regulatory factor, participates in the occurrence and development of RA through diverse pathways (4). PIWI-interacting RNA (piRNA, 24–32 nt), compared with microRNA (miRNA), long non-coding RNA (lncRNA), etc., is a relatively unexplored ncRNA that is characterized by specific interaction with PIWI protein, a member of the Argonaute family, to form an RNA-induced silencing complex to protect genome integrity by suppressing transposable elements (TEs) (5). In fact, in addition to negative regulation, the piRNA/PIWI system can also stabilize the expression of target genes and induce translational activation according to different binding sites (6). Diversified functional regulation modes also show great research potential. Since piRNA was initially mainly found in mammalian germ lines and embryonic stem cells (7, 8), most of the early studies on piRNA were focused on this (9, 10). Nowadays, with the expansion of the research scope, piRNA has been gradually found in the heart, islet, nervous system, and other somatic cells (11–13) and is closely associated with various diseases, such as breast cancer, hepatocellular carcinoma, bladder cancer, multiple myeloma, and so on (14–20). Elena et al. identified that many piRNAs were expressed differentially after lineage commitment towards osteo- and chondrogenesis of human bone marrow mesenchymal stromal cells (hMSCs) (21). Zhong et al. found that piR30840 interacts with Piwil4 and Ago4 and targets the intron of the IL-4 gene, resulting in decreased IL-4 secretion from CD4+T cells and inhibiting Th2 cell differentiation (22). Furthermore, similar to miRNA, piRNA can be detected in easily accessible samples (such as serum, plasma, saliva, and urine) (23) and remain stable regardless of repeated freeze-thawing or long-term incubation, which has the potential to be a disease biomarker (24). Pleštilová et al. reported that the piRNA/PIWI system exists in synovial tissues and synovial fibroblasts of RA patients and might be involved in signaling pathways for host defense and inflammation (25). However, whether piRNAs are indeed associated with RA remains unclear. We also do not know if piRNA dysregulation occurs in patients’ peripheral blood (which is an easier sample to obtain than synovial tissue), except for synovial tissue.

Accordingly, this study aims to reveal the expression of piRNA/PIWI in peripheral leukocytes of RA patients and their correlation with clinical indexes, and preliminarily explore potential mechanisms based on bioinformatics analysis, which will provide preliminary research data for further exploration of the role of the piRNA/PIWI system in the RA pathogenesis.

## 2 Materials and methods

### 2.1 Patients and samples

All new-onset untreated RA patients and healthy controls (HCs) were recruited from the West China Hospital of Sichuan University between October 2020 and March 2023. The inclusion criteria for new-onset untreated patients were fulfilled with the ACR/EULAR 2010 Classification Criteria for Rheumatoid Arthritis (26) and diagnosed at West China Hospital. The exclusion criteria were as follows: treated with immunosuppressive agents, glucocorticoids, and DMARDs before diagnosis; long-term smoking or passive smoking history; and suffering from other autoimmune diseases, acute co-infections, cancers, and other systemic chronic diseases simultaneously. During the same period, gender- and age-matched healthy volunteers were enrolled in the control group. All blood samples were collected before drug treatment. DAS28-CRP, anti-cyclic citrullinated peptide antibody (ACPA), C-reactive protein (CRP), rheumatoid factor (RF), and many other clinical data were collected when patients were recruited. This study was approved by the Ethics Committee of the West China Hospital of Sichuan University.

### 2.2 RNA extraction

Total RNA was extracted from peripheral leukocytes using QIAzol^®^ Lysis Reagent (Qiagen, Hilden, Germany) and purified by the miRNeasy mini-Kit (Qiagen, Hilden, Germany). The concentration and purity of the RNA were assessed by a NanoVue Plus microspectrophotometer (GE Healthcare, Madison, WI, USA).

### 2.3 Small RNA sequencing and bioinformatic analysis

Three RA patients and three HCs were selected for detecting small RNAs with small RNA sequencing. The RNA quality was assured with an Agilent 2100 chip (Agilent Technologies, USA). Small RNA library preparations and sequencing were conducted at Shanghai Oebiotech Co. (Shanghai, China).

The basic reads were converted into raw data by base calling. Low-quality reads were filtered, and the reads with 5’ primer contaminants and poly (A) were removed. The reads without the 3’ adapter and insert tag, the reads shorter than 15 nt and longer than 41 nt from the raw data, were filtered, and the clean reads were obtained. The clean reads from other known types of small RNAs (rRNAs, tRNAs, snRNAs, snoRNAs, and miRNAs) were removed by aligning them to Rfam v.10.1 (http://www.sanger.ac.uk/software/Rfam) (27), miRBase databases (https://www.mirbase.org/) (28), and GenBank databases (http://www.ncbi.nlm.nih.gov/genbank/). Through the above several methods to filter, obtained clean reads were aligned with the known piRNA from the piRBase (http://www.regulatoryrna.org/database/piRNA/) (29) with Bowtie software without mismatches.

The piRNA expression levels of each sample were normalized using TPM. Differentially expressed (DE) piRNAs were identified with the threshold of fold change >2 and *P <*0.05, calculated with the DEG algorithm (30) in the R package for experiments with biological replicates. The targets of DE piRNAs were predicted by Miranda software and piRNAQuest V.2 (31), with the parameters as follows: S ≥ 150, ΔG ≤ −30 kcal/mol. Gene ontology (GO) enrichment and Kyoto Encyclopedia of Genes and Genomes (KEGG) pathway enrichment analysis of DE piRNA-target- gene were respectively performed using R based on the hypergeometric distribution.

### 2.4 Analysis of gene profile targeted by immunoregulation-related piRNA

The GEO database was used to obtain transcriptome sequencing data. Screening criteria included the following: (1) *Homo sapiens* expression profiling by high throughput sequencing; (2) peripheral blood of RA patients or synovial tissue from joint synovial biopsies; (3) datasets contain no less than five samples. Finally, two datasets, GSE193193 and GSE 77298, were selected for further analysis (**Table Supplementary 1**). The statistical programming language R (version 4.0.3) (http://www.r-project.org/) was used for log2 transformation of the data. The “SVA” package was used for batch correction. The R package “limma” was used to identify differentially expressed genes (DEGs), which were defined as the absolute value of log2 fold change |log2FC| >1 and adjusted *P <*0.05. To better visualize these genes, the pheatmap package was used to make heatmaps.

### 2.5 Quantitative reverse transcriptase-polymerase chain reaction (RT-qPCR)

To verify the expression levels of immunoregulation-related piRNA identified from RA patients by RNA sequencing, five upregulated piRNAs (piR-hsa-27620, piR-hsa-27124, piR-hsa-23940, piR-hsa-27400, and piR-hsa-30353) and two downregulated piRNAs (piR-hsa-25672 and piR-hsa-4946) were selected and validated with the All-in-One™ miRNA qRT-PCR Detection Kit (GeneCopoeia, Rockville, MD, United States). All-In-One 5× RT MasterMix and BlasTaq™ 2× qPCR MasterMix (ABM, Vancouver, BC, Canada) were used to estimate the expression of PIWI and other proteins implicated in the piRNA pathway.

Template RNA was reverse transcribed into cDNA in a Veriti 96-Well Thermal Cycler (Applied Biosystems, Forster City, CA, USA) and measured by an ultraviolet spectrophotometer (Eppendorf, Hamburg, Germany). Real-time PCR was performed on the QuantStudio^®^Q5 Real-Time PCR system (Applied Biosystems). NRT (no reverse transcriptase) and NTC (no template control) served as negative controls. U6 and GAPDH served as the reference genes. The relative expression level was calculated by the 2^−ΔΔCT^ method. All primers (**Table S2**) were synthesized by Sangon Biotech (Shanghai) Co., Ltd.

### 2.6 Statistical analysis

All data were analyzed using SPSS (version 26.0; SPSS Inc., Chicago, IL, USA), SAS (version 9.4; SAS Institute, Cary, NC), and GraphPad Prism v9.0 (GraphPad Software, San Diego, CA, USA) software. When the clinical data were lower than the limit of detection, the detection limit was used instead of the true value. For quantitative data, the Shapiro–Wilk normality test was used for normality analysis. The normal distribution data was presented by mean ± standard deviation and analyzed by the Student-t test. Non-normally distributed data were represented by the median (interquartile spacing) [M(IQR)] and analyzed by the Mann–Whitney test. Categorical data were evaluated using the Chi-square test. Logistic regression analysis and receiver operating characteristic (ROC) curves were generated to determine the value of piRNAs as diagnostic biomarkers. Spearman’s correlation (*r_s_*) was used to analyze the correlation of candidate piRNAs with the levels of clinical data. A difference of *P <*0.05 was considered statistically significant. G*Power was used to estimate the sample size and compute statistical power (32). A power greater than 0.8 was considered acceptable.

## 3 Results

### 3.1 Characteristics of the study population

The clinical information of the studied patients is presented in **Table 1**. There were no differences in age (*P* = 0.136) or gender (*P* = 0.360). The RA laboratory diagnostic index, ACPA, RF, and AKA of patients were significantly higher than those of healthy individuals (all *P <*0.001). Patients had elevated CRP and C3 levels, indicating active inflammation. Furthermore, the blood routine of RA patients showed significantly different patterns compared with that of healthy individuals.

**Table 1:** Demographic and clinical characteristics of the study participants.

Index	RA (n = 45)	HC (n = 84)	*p*-value
Age (years)	45.19 ± 13.18	41.73 ± 11.54	0.136
Gender (female/male)	35/10	59/25	0.360
Disease duration (month)	7.50 (2.00–12.00)	–	–
ACPA (U/ml)	469.50 (170.75–500.00)	8.00 (8.00–8.00)	**<0.001**
RF (IU/ml)	143.50 (47.90–319.50)	20.00 (20.00–20.00)	**<0.001**
CRP (mg/L)	4.94 (2.74–13.83)	1.61 (1.28–2.15)	**<0.001**
AKA (positive/negative)	34/11	0/66	**<0.001**
DAS28-CRP	4.31 (2.69–5.54)	–	–
WBC (10^9^/L)	6.89 ± 1.89	5.29 ± 1.04	**<0.001**
NEUT (%)	66.44 ± 9.52	57.14 ± 8.26	**<0.001**
LYMPH (%)	24.62 ± 8.38	32.94 ± 7.53	**<0.001**
MONO (%)	6.65 ± 1.63	7.00 ± 1.64	0.266
NEUT (10^9^/L)	4.65 ± 1.72	3.05 ± 0.88	**<0.001**
LYMPH (10^9^/L)	1.63 ± 0.56	1.71 ± 0.43	0.431
MONO (10^9^/L)	0.45 ± 0.14	0.37 ± 0.10	**0.001**
RBC (10^12^/L)	4.46 ± 0.41	4.61 ± 0.44	0.072
HGB (g/L)	130.64 ± 15.57	141.28 ± 14.26	**<0.001**
HCT (L/L)	0.41 ± 0.04	0.43 ± 0.04	**<0.001**
MCV (fl)	90.96 ± 5.97	93.46 ± 3.80	**0.005**
MCH (pg)	29.31 ± 2.57	30.66 ± 1.42	**0.003**
MCHC (g/L)	321.79 ± 11.03	328.01 ± 8.31	**0.001**
RDW-CV (%)	13.10 (12.58–13.90)	12.80 (12.50–13.25)	0.068
PLT (10^9^/L)	231.10 ± 73.67	214.36 ± 49.57	0.190
IGG (g/L)	15.84 ± 3.16	11.77 ± 0.55	**0.037**
IGA (mg/L)	3,214.62 ± 1,308.45	2,470.00 ± 738.99	0.347
IGM (mg/L)	1,754.65 ± 743.50	969.33 ± 278.43	0.085
IGE (IU/ml)	74.03 ± 89.86	69.84 ± 97.36	0.946
C3 (g/L)	1.03 ± 0.22	0.76 ± 0.09	**0.030**
C4 (g/L)	0.24 ± 0.07	0.18 ± 0.03	0.122

Data are presented as mean ± standard deviation, median, and median (interquartile range) [M (IQR)]. Comparisons between groups were made by the Student-t test, Mann–Whitney test or Chi-square test as appropriate. P <0.05 is considered of statistical significance (boldface type). RA, rheumatoid arthritis; HC, healthy control; ACPA, anti-cyclic citrullinated peptide antibody; CRP, C-reactive protein; RF, rheumatoid factor; AKA, anti-keratin antibody; Other abbreviations will be presented in the **supplementary material**.

### 3.2 The piRNA dysregulation profile was revealed by small RNA sequencing in peripheral leukocytes of RA patients

**Figure 1:**
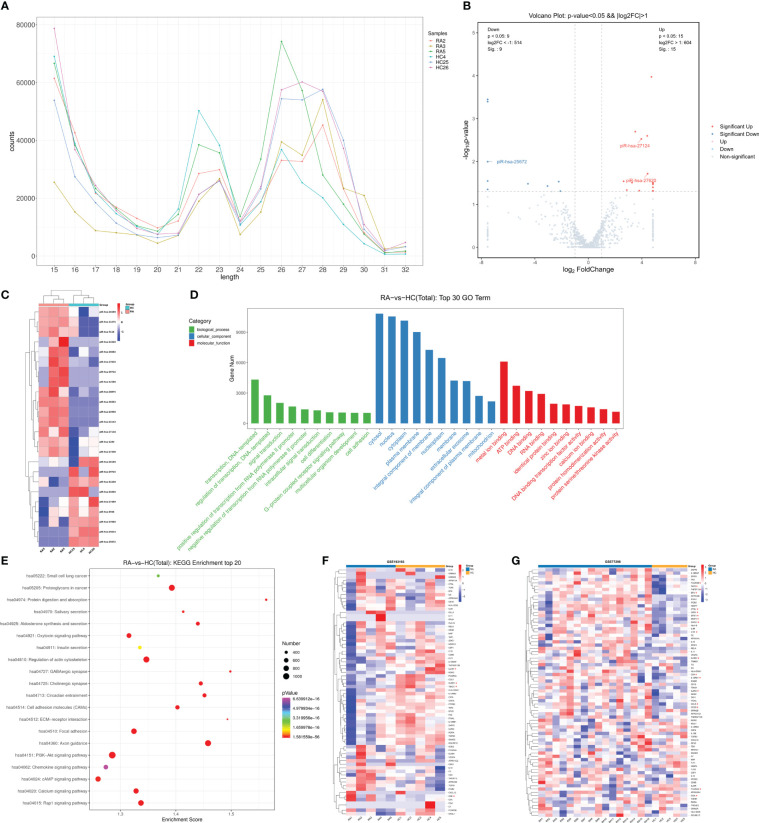
Small RNA sequencing revealed piRNA dysregulation in peripheral leukocytes of RA patients. **(A)** The known piRNA length distribution line graph. **(B)** The volcano plot. The gray point indicated no difference in piRNA between groups, red points represented upregulated expression, and blue represented downregulated expression significantly. **(C)** Clustering of expression patterns of 24 differentially expressed piRNAs. Each row represents one piRNA, and each column represents a sample. Red, upregulation; blue, downregulation. HC4, HC25, and HC26, healthy individuals, RA2, RA3 and RA5, RA patients. **(D)** The top 10 terms of the GO annotation analysis of genes targeted by dysregulated piRNAs. **(E)** The top 20 terms of KEGG enrichment analysis. The ordinate represents gene-related pathways, the abscissa represents enrichment score, and the size of the dots represents the number of genes. The lower the p-value, the redder the color tends to be. **(F)** The heat map showing profiles of genes targeted by immunoregulation-related piRNAs in the peripheral blood of RA patients (GSE193193). The asterisk (*) indicates significant differences, |log2FC| >1 and adjusted *P <*0.05. **(G)** The heat map showing profiles of genes targeted by immunoregulation-related piRNAs in synovial tissue of RA patients (GSE77298). * |log2FC| >1 and adjusted *P <*0.05.

To comprehend the patterns of piRNA expression, small RNA sequencing was used to explore the small RNA expression profiles in the peripheral leukocytes of healthy people and RA patients. Sequence alignment annotation results of six blood samples showed that a total of 1,565 known piRNAs were identified. The length distribution displayed two different peaks, one at the 21–23 nt position, corresponding to miRNA in size, and another concentrated in the 26–28 nt position, corresponding to piRNA in size (**Figure 1A**). Furthermore, to explore the differential expression of piRNA in RA patients and healthy controls, we conducted expression analysis with the DEG algorithm. The box– whisker plot indicated no abnormal expression in the six samples (**Figure S1**). Then, after the piRNA expression levels of the two groups were standardized, we used fold change ≥ 2 and *P* < 0.05 as the standard to screen DE piRNAs. The overall picture of piRNA expression between the two groups was displayed in the volcano plot (**Figure 1B**). Hierarchical clustering revealed the expression of DE piRNAs in each sample (**Figure 1C**). A total of 24 DE piRNAs were recognized, including nine downregulated and 15 upregulated piRNAs (**Table S3**).

### 3.3 GO term and KEGG pathway enrichment analysis of DE piRNAs

Target genes of the DE piRNAs were calculated by the Miranda algorithm and piRNAQuest V.2, and 48,908 genes were predicted. GO and KEGG pathway analyses were performed on target genes to investigate the role of differentially expressed piRNA in RA pathogenesis.

Based on the enriched dysregulated piRNA targets originated from the gene annotation, the top 10 GO classifications of biological process (BP), cell components (CC), and molecular function (MF) were shown according to routine GO classification algorithms (**Figure 1D**). In BP, target genes of DE piRNAs were significantly enriched in transcription, regulation of transcription, signal transduction, regulation of transcription from the RNA polymerase II promoter, cell differentiation, the G- protein-coupled receptor signaling pathway, and cell adhesion. In CC, they were mainly concentrated on the cytosol, nucleus, cytoplasm, nucleoplasm, extracellular exosome, and mitochondrion. In MF, genes were primarily related to metal ion binding, ATP binding, DNA binding, RNA binding, identical protein binding, zinc ion binding, and so on. The top 10 GO processes of upregulated/downregulated piRNA targets were also enriched in the regulation of transcription, signal transduction, cell differentiation, and cell adhesion in the BP subgroup (**Figures S2A, B**).

KEGG pathway analysis assists us in better understanding the biological function of genes. **Figure 1E** illustrates the top 20 ranked significant pathways for DE piRNA targets in KEGG. The targets of upregulated piRNAs were primarily enriched in the PI3K–Akt signaling pathway, Rap1 signaling pathway, cAMP signaling pathway, axon guidance, regulation of the actin cytoskeleton, calcium signaling pathway, proteoglycans in cancer, and so on (**Figure S2C**). Downregulated piRNA targets were also enriched in many important pathways, such as the PI3K– Akt signaling pathway, MAPK signaling pathway, Th17 cell differentiation, cGMP–PKG signaling pathway, and ECM–receptor interaction (**Figure S2D**).

Subsequently, to screen piRNAs that may be related to immune regulation, GO and KEGG enrichment analyses of target genes of the 24 DE piRNAs were performed, and the target genes enriched in the immune system, immune signaling pathways, and immune diseases were screened. Based on the target gene enrichment analysis, piRNAs that regulate those target genes were screened. A total of seven piRNAs highly correlated with RA immune regulation were selected for subsequent RT-qPCR verification, and the screening results are shown in **Table S4.**

### 3.4 The expression of gene targets of immunoregulation-related piRNAs

To better understand the expression of genes that were potentially regulated by immunoregulation-related piRNA, two datasets (GSE193193 and GSE77298) were selected from the GEO database to analyze their expression in the peripheral blood and synovial tissue of RA patients, respectively. GSE193193, including five peripheral blood mononuclear cells (PBMCs) from RA patients and five PBMCs from HCs, was used to analyze the expression profile of genes targeted by immunoregulation-related piRNAs in the peripheral blood of RA patients (**Figure 1F**, **Table S5**). *KLRD1*, *TBX21*, and *IL23R*, the potential targets of piR-hsa-25672, piR-hsa-27620, and piR-hsa-23940, respectively, were all significantly downregulated, while the level of *C4B*, the potential target of piR-hsa-25672, was significantly higher in RA patients than that in HCs. The GSE77298 dataset was used to explore these gene profiles in synovial tissue (**Figure 1G**). Differential expression analysis showed that 18 genes (*STAT1*, *TNFSF13*, *MMP3*, *FCGR2A*, *CCL5*, etc.) were significantly differentially expressed in synovial tissue (**Table S6**).

### 3.5 Validation of immunoregulation-related piRNAs with RT-qPCR

**Figure 2:**
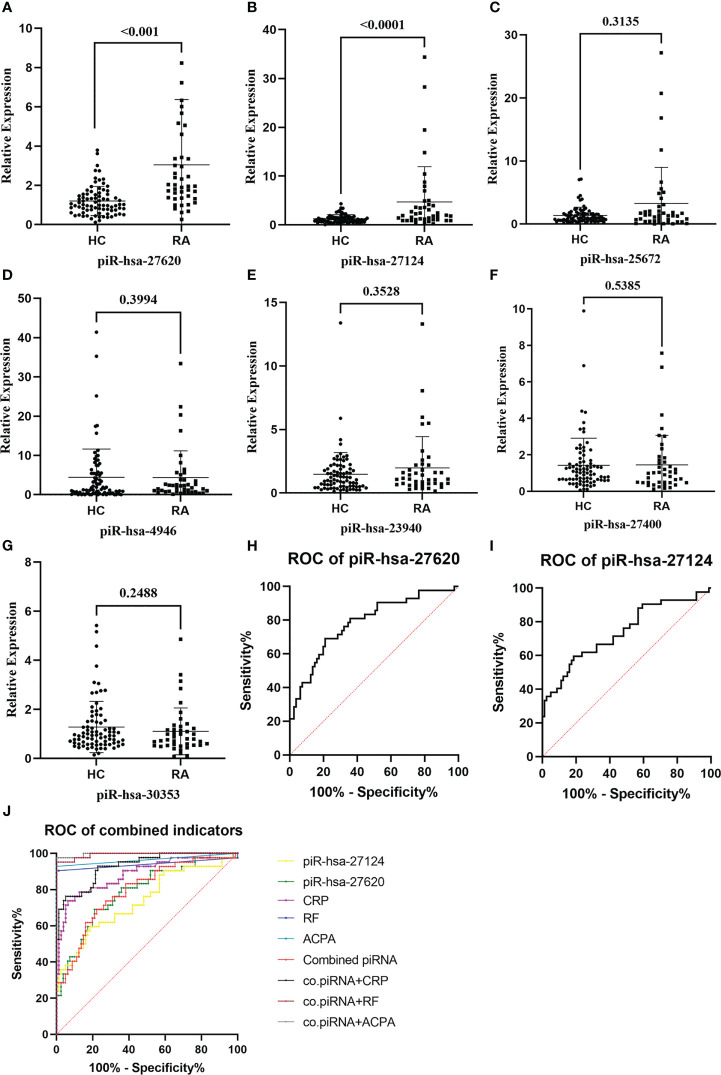
The expression levels of immunoregulation-related piRNAs were validated, and some of them had good abilities to distinguish patients with rheumatoid arthritis from healthy individuals. **(A–G)** Seven piRNAs were selected and evaluated by RT-qPCR in 37 new-onset RA patients and 63 HCs. Two piRNAs, [piR-hsa-27620 (*P* < 0.001) and piR-hsa-27124 (*P* < 0.001)], were significantly higher in RA patients. **(H, I)** Diagnostic evaluation of those two immunoregulation-related piRNAs assessed by the ROC curve. **(J)** Logistic regression analysis was used to synthesize the two differentially expressed piRNAs to construct the combined piRNA, which was then compared with RA diagnostic markers (RF, anti-CCP, and CRP).

RT-qPCR was performed to validate the expression levels of seven candidate piRNAs in 42 new-onset RA patients and 81 HCs (**Table 2**). According to the qPCR results, the levels of two piRNAs [piR-hsa-27620 (*P* < 0.001, power = 0.98) and piR-hsa-27124 (*P* < 0.001, power = 0.95)] were significantly higher in RA patients than those in HCs. Nevertheless, no significant difference was observed in the other five piRNAs (**Figures 2A–G**).

**Table 2:** piRNA expression levels in patients with RA and healthy controls.

piRNA	RA [M (IQR)]	HC [M (IQR)]	*P*-value
piR-hsa-27620	1.9540 (1.3400–3.3740)	1.0180 (0.7252–1.5460)	**<0.001**
piR-hsa-27124	1.9380 (1.0690–4.3570)	1.0680 (0.6131–1.5300)	**<0.001**
piR-hsa-25672	1.3650 (0.4218–2.8570)	0.9721 (0.5551–1.6410)	0.314
piR-hsa-4946	1.9800 (0.7675–4.1480)	1.3490 (0.3879–5.4720)	0.399
piR-hsa-23940	1.1000 (0.6975–2.2000)	1.0420 (0.5068–1.8970)	0.353
piR-hsa-27400	1.0050 (0.4700–1.7430)	1.0072 (0.6410–1.4548)	0.539
piR-hsa-30353	0.7950 (0.5375–1.2400)	1.0520 (0.6296–1.6070)	0.249

P <0.05 is considered of statistical significance (boldface type). Comparisons between groups were made by Mann–Whitney test. U6 served as the reference gene.

### 3.6 Evaluation of the diagnostic value of immunoregulation-related piRNAs

With the purpose of understanding the ability of immunoregulation-related piRNAs to distinguish between RA and HC, ROC curves were generated to identify the diagnostic value of those two piRNAs (**Figures 2H–J**). The AUC values of piR-hsa-27620 and piR-hsa-27124 were respectively 0.7851 [95% CI (0.6982, 0.8721)] and 0.7390 [95% CI (0.6416, 0.8363)], which both had high sensitivity (79.00% and 81.50%, respectively) and specificity (69.00% and 59.50%, respectively) when the largest Youden’s index was the optimal operating point (**Table 3**). Logistic regression analysis with ROC curves was used to construct the combined indicators. Compared with the discriminative ability of clinical indicators, piRNA alone has insufficient diagnostic ability; however, when combining piRNAs with other indicators, the discriminative ability of the combination index can exceed that of any single clinical indicator.

**Table 3:** Diagnostic values of immunoregulation related piRNAs and clinical indicators.

piRNA	AUC (95% CI)	*P*-value	Youden’s index	SEN	SPE	PLR	NLR
piR-hsa-27620	0.7851 (0.6982, 0.8721)	<0.0001	0.4800	0.7900	0.6900	2.5484	0.3043
piR-hsa-27124	0.7390 (0.6416, 0.8363)	<0.0001	0.4100	0.8150	0.5950	2.0123	0.3109
Combined piRNAs†	0.7925 (0.7080, 0.8770)	<0.0001	0.4680	0.7780	0.6900	2.5097	0.3217
ACPA	0.9643 (0.9185, 1.000)	<0.0001	0.9290	0.9290	1	–	0.0710
RF	0.9400 (0.8761, 1.000)	<0.0001	0.9050	0.9050	1	–	0.0950
CRP	0.8874 (0.8218, 0.9531)	<0.0001	0.6750	0.9370	0.7380	3.5763	0.0854
Combined piRNAs + ACPA	0.9965 (0.9892, 1.000)	<0.0001	0.9760	0.9760	1	–	0.0240
Combined piRNAs + RF	0.9932 (0.9828, 1.000)	<0.0001	0.9520	0.9520	1	–	0.0480
Combined piRNAs + CRP	0.9289 (0.8827, 0.9751)	<0.0001	0.7110	0.9490	0.7620	3.9874	0.0669

†Combined piRNAs include piR-hsa-27620 and piR-hsa-27124. SEN, sensitivity; SPE, specificity; PLR, positive likelihood ratio; NLR, negative likelihood ratio. P ≤0.05 was considered statistically significant.

### 3.7 PIWIL protein profiles in the peripheral blood of RA patients and HC

**Figure 3:**
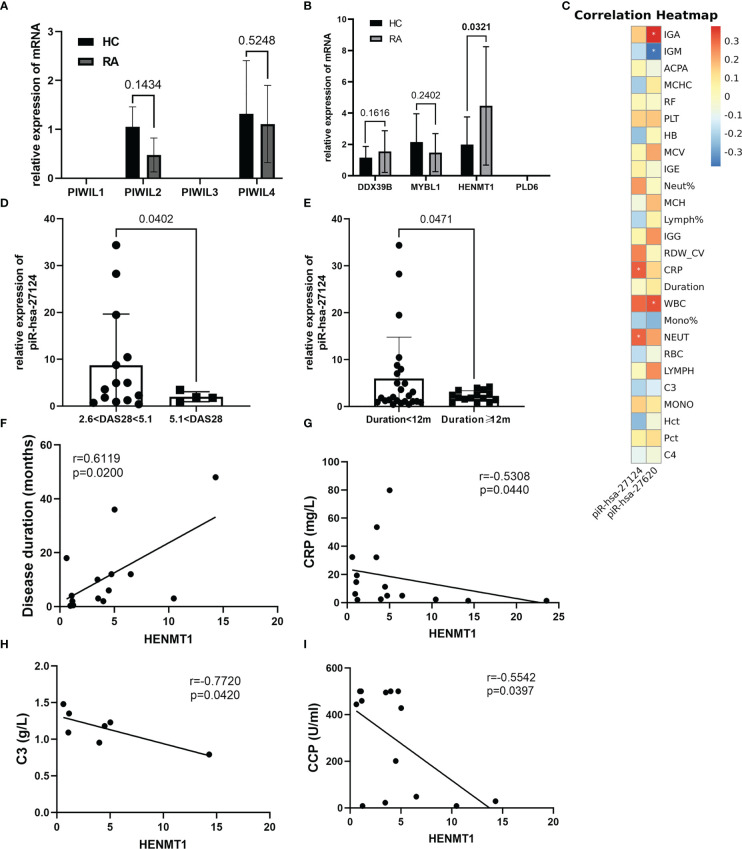
The piRNA/PIWI system operates in the peripheral blood of RA patients and may participate in the pathogenesis of RA. **(A)** The expression of PIWIL1-4 mRNA was estimated by RT-qPCR. Both PIWIL2 and PIWIL4 expressed but had no significant difference between the two groups. **(B)** Proteins implicated in the piRNA pathway were first selected by public databases and then validated by RT-qPCR. Although PLD6 was not expressed, the functions of the other three proteins already covered the entire process of piRNA production, transfer, and processing. **(C)** The correlation heatmap showed the association between two immunoregulation-related piRNAs and clinical indicators. Red and blue colors represent negative and positive correlations, respectively. *p <0.05. **(D, E)** Further stratified analysis revealed that piR-hsa-27124 was significantly reduced in patients with disease activity greater than 5.1 and a disease course longer than 1 year, although there was no direct correlation between them. **(F–I)** Clinical indicators that were significantly associated with HENMT1 mRNA level.

The PIWI proteins belong to the Argonaute family, which has crucial functions in piRNA biogenesis and functional complex formation. We first measured the expression of the PIWIL1-4 mRNA. PIWIL2 and PIWIL4 were expressed similarly in RA and HC, while the mRNAs of PIWIL1 and PIWIL3 were not detectable (**Figure 3A**). In addition, we also examined genes (DDX39B, MYBL1, HENMT1, and PLD6) related to piRNA metabolism and found that HENMT1 was significantly overexpressed in RA patients (**Figure 3B**).

### 3.8 Correlation between piRNAs/PIWI system and RA clinical characteristics

Our data have suggested that the expression levels of piR-hsa-27620, piR-hsa-27124, and HENMT1 were elevated in the peripheral blood of RA patients. To further study their association with disease characteristics, we performed correlation analysis and presented the groups with significant correlations in the form of heatmap and scatter plots (**Figure 3C**, **Figure S3**). The results showed that piR-hsa-27620 was positively correlated with IgA (*r_s_* = 0.4616, *P* = 0.0176), while negatively correlated with IgM (*r_s_* = −0.5133, *P* = 0.0087). Furthermore, piR-hsa-27124 was positively correlated with neutrophil count (*r_s_* = 0.3069, *P* = 0.0481), and CRP levels (*r_s_* = 0.3190, *P* = 0.0395). Furthermore, despite the lack of direct correlation between disease duration, DAS28-CRP, and piRNAs expression, we discovered through stratified analysis that piR-hsa-27124 dramatically decreased in patients with high disease activity and disease duration of more than 1 year (**Figures 3D, E**).

HENMT1, a methyltransferase that adds a 2’-O-methyl group at the 3’-end of piRNAs, increased gradually over the course of the disease (*r_s_* = 0.6119, *P* = 0.0200) and was negatively correlated with CRP, C3, and anti-CCP (*r_s_* = −0.5308, *P* = 0.0440; *r_s_* = −0.7720, *P* = 0.0420; *r_s_* = −0.5542, *P* = 0.0397, respectively) (**Figures 3F–I**).

## 4 Discussion

In this study, 1,565 piRNAs were identified in the peripheral leukocytes of RA patients from southwest China by small RNA sequencing, and 24 piRNAs were differentially expressed significantly. Then the GO term and KEGG pathway enrichment analysis were conducted, and seven immunoregulation-related piRNAs were selected; among them, piR-hsa-27620 and piR-hsa-27124 were not only significantly upregulated in patients with rheumatoid arthritis but also may be involved in immune regulation, which has potential research value as biological markers of RA.

RA is a chronic inflammatory rheumatoid disease mediated by immunity, which will eventually cause disabling joint destruction and is closely related to genetic background. The piRNA/PIWI system has long been reported to be involved in epigenetic regulation (5, 6, 33), which is also associated with RA (34–36). Transcriptome sequencing data show that piRNA clusters exist in a variety of immune cells related to inflammatory reactions, such as neutrophils, macrophages, dendritic cells, B cells, CD4+T cells, and NK cells (37–39). Therefore, we analyzed and validated that the piRNA/PIWI system is dysregulated in the peripheral leukocytes of RA.

Studies have found that piRNAs may be the largest class of non-coding RNAs and have more regulation modes compared with other ncRNAs (6, 40), which means that the biological processes affected by dysregulated piRNAs will be more extensive in the pathogenesis of diseases. Our sequencing results also showed that the number of fragments in the piRNA region at 26–28 nt was higher than in other regions. Bioinformatics analysis showed that DE piRNAs might participate in the transcriptional regulation of various RA regulatory molecules, such as *STAT3*, *FCGR2A*, *CXCL12*, etc. GO analysis results showed that piRNA might intervene in the disease by participating in transcriptional regulation, intracellular signal transduction, RNA polymerase II promoter transcriptional regulation, cell differentiation, and other processes, indicating that the whole functional system of piRNAs exists in RA and may change in response to disease status. KEGG pathway analysis results showed that piRNA may be involved in many RA-related signaling pathways, including the Rap1 signaling pathway, the PI3K–Akt signaling pathway, the MAPK signaling pathway, Th17 cell differentiation, apoptosis mediated by natural killer cells, and osteoclast differentiation. These are all available in **Table S4**. Therefore, we can speculate that piRNA may be involved in the pathogenesis of RA through transcription and post-transcriptional regulation, affecting immune cell differentiation and the secretion of inflammatory factors. Additionally, according to the GEO database, we analyzed the expression of genes targeted by immunoregulation-related piRNAs in the peripheral blood and synovial tissues of RA patients. The results showed that many gene targets are dysregulated in RA patients, which suggests indirectly the potential function of dysregulated piRNAs. Furthermore, it has been reported that the piRNA/PIWI system can also stabilize the expression of target genes and induce translational activation according to the different binding sites, in addition to negative regulation (6). That is to say, the expression of gene targets regulated by piRNAs does not always exhibit an inverse trend to that of the piRNAs themselves. Similarly, we also observed that upregulated piRNAs may target upregulated genes, for instance, piR-hsa-27620, *STAT1*, piR-hsa-27400, and *TNFSF13*. Of course, it is still imperative to conduct clinical studies on the same patient population and molecular mechanisms to obtain direct evidence, which will help illustrate the position of immunoregulation-related piRNAs in the inflammatory pathway.

Since discovering that the expression of piRNA is not limited to germ cell lines and could be used for auxiliary diagnosis and monitoring of diseases (23, 24), more and more studies have demonstrated this point of view. Mai et al. demonstrated that piR-hsa-54265 is an oncogenic RNA in the development of colorectal cancer (CRC) and a valuable biomarker better than other serum tumor markers routinely used in clinic, which would decrease dramatically after surgical resection of CRC but increase again when tumor relapses (41). Lipps et al. identified the piRNA DQ593039 as a promising biomarker reflecting the severity of pulmonary hypertension (42). The results of transcriptome-wide piRNA profiling revealed that up to 103 piRNAs nominally correlated with genome-wide significant risk SNPs for Alzheimer’s disease (AD) (43). With these studies, our research demonstrated that piRNA expression is indeed not limited to germlines and might have a potential correlation with rheumatoid arthritis. The ROC curve built with 42 RA patients and 81 controls yielded that piR-hsa-27620 had the best diagnostic accuracy [AUC (95% CI): 0.7851 (0.6982, 0.8721), SEN: 79.00%, SPE: 69.00%], while piR-hsa-27124 had the highest sensitivity [AUC (95% CI): 0.7390 (0.6416, 0.8363), SEN: 81.50%, SPE: 59.50%], which have good abilities to distinguish patients with rheumatoid arthritis from healthy individuals.

To better demonstrate the landscape of the piRNA/PIWI system in RA peripheral blood, we assessed the expression of PIWI protein and piRNA biogenesis-related enzymes subsequently. Similar to the synovial tissue of RA (25), only PIWIL2 and PIWIL4 expressions were detected in the peripheral blood of RA. Due to the large number of enzymes involved in piRNA biogenesis (5), we first observed their expression in peripheral blood based on preliminary sequence data from public databases, and then selected four representative enzymes for further verification based on their functional roles in piRNA processing. Among them, MYBL1 can promote transcription of piRNA clusters, DDX39B is important for enabling nuclear export of piRNA precursor transcripts to sites of piRNA production, HENMT1 is required to modify the 2’ hydroxyl at the piRNA 3’ end, and PLD6 participates in establishing the 3’ ends of responder pre-piRNAs. Our findings suggested that except for PLD6, the other three enzymes were all expressed in RA peripheral blood, and HENMT1 was significantly elevated. Interestingly, studies have already found that DDX39B is associated with RA susceptibility independently of HLA-DRB1 (44), further strengthening the evidence of the relationship between piRNAs and RA. Moreover, though PLD6 was not detectable in RA, it is reported that carrying HLA-B*27 was associated with robust hypomethylation of PLD6 in ankylosing spondylitis patients (45). Afterward, correlation analysis was conducted to better align these results with clinical information. According to our results, neutrophil, an essential immune cell in modulating autoimmunity (46), may have more piR-hsa-27124 and piR-hsa-25672. Positively correlated with IgA level, piR-hsa-27620 might be involved in RA mucosal immunity. Besides, piR-hsa-27124 was associated with inflammation, and patients with moderate symptoms in the early stages of RA exhibited higher expression levels than those with more severe symptoms and longer disease courses. Meanwhile, HENMT1, necessary for piRNA stability, presented an increasing trend with the duration of RA, implying that the piRNA/PIWI system might initiate the stabilization mechanism in response in the later stages of the disease.

The piRNA/PIWI system expression profile in the peripheral leukocytes of RA patients was discussed and verified for the first time in our project, and it was found that the piRNA/PIWI system was dysregulated and that two immunoregulation-related piRNAs, i.e., piR-hsa-27620 and piR-hsa-27124, had value as potential biomarkers to help improve the diagnostic ability of current indicators. This project also had some limitations. First, we only explored the landscape of the piRNA/PIWI system in new-onset untreated RA patients; therefore, further exploration is required to determine the impact of clinical treatment on this system. Second, due to the limitations of current studies on the function of piRNA, this study only made superficial preliminary assumptions through bioinformatics analysis and comparison with clinical indicators and could not clarify the role of piRNAs in the pathogenesis of RA in detail. The deficiencies of the current study point to the direction of our next experiment. It is believed that with the expansion of the breadth and depth of the study, the function of immunoregulation-related piRNAs will be revealed gradually.

## Data availability statement

The sequence data reported in this paper have been deposited in the Genome Sequence Archive (Genomics, Proteomics & Bioinformatics 2021) in National Genomics Data Center (Nucleic Acids Res 2022), China National Center for Bioinformation / Beijing Institute of Genomics, Chinese Academy of Sciences (GSA-Human: HRA004568) that are publicly accessible at https://ngdc.cncb.ac.cn/gsa-human.

## Ethics statement

The studies involving human participants were reviewed and approved by Ethics Committee of West China Hospital, Sichuan University. The patients/participants provided their written informed consent to participate in this study.

## Author contributions

BY and YW designed this study and contributed to the research idea. RR, HT, and ZH contributed to the research data and wrote this manuscript. BY, YW, and RR revised and edited this manuscript. BY was the guarantor of this work, and as such, had full access to all the data in the study and takes responsibility for the integrity of the data and the accuracy of the data analysis. All authors listed have made a substantial, direct, and intellectual contribution to the work and approved it for publication.
